# Survey data of a traditional communal water irrigation system in Northern Thailand

**DOI:** 10.1016/j.dib.2022.108515

**Published:** 2022-08-05

**Authors:** Arriya Mungsunti, Kevin A. Parton, Arief Anshory Yusuf, Tossapond Kewprasopsak

**Affiliations:** a Center for Sustainable Development Goals Studies (SDGs Center), Padjadjaran University, Bandung 40132, Indonesia; b Gulbali Institute, School of Business, Charles Sturt University, Bathurst, NSW 2795, Australia; c Faculty of Economics, Chiang Mai University, Chiang Mai, Thailand

**Keywords:** *Muang fai*, Common pool resources, Ostrom principles, Surface irrigation

## Abstract

This data article contains a description of a dataset collected by a survey on a traditional communal water irrigation system. This is the *Muang fai*, a 700-years old communal irrigation system in Northern Thailand. The *Muang fai* is managed through a series of regulations that are close to Ostrom's principles of effective common property resources (Ostrom, 1990). The survival of this long-standing practice, including its knowledge of the water-flow characteristics of the watershed, is under threat as new technologies, such as groundwater pumping, become increasingly accessible. The target population of the survey was the group of Longan farmers who are located within the 12 villages that are engaged in *Muang fai* Sop Rong in Chiang Mai Province, Northern Thailand. Information was specifically collected about irrigation practices, farmland characteristics and socio-economic variables from 570 longan (their main crop) farmer households. Roughly half of these sampled farmers practise *Muang fai*, the other half practise underground pumping irrigation. The irrigation information collected includes type of irrigation (surface or underground), the quantity of water used, pumping methods (if pumps are used). Farming characteristics collected include volume and value of the harvests, land size, and distance to irrigation canal. Socioeconomic characteristics included among others: farmers’ income, expenditure, education, off-farm employment and *Muang-fai* membership. This dataset can be a source of baseline information for future research as well as help preserve the knowledge of this tradition.

## Specifications Table

**Table:** 

Subject	Agricultural economics
Specific subject area	Irrigation, farming and socio-economic characteristics of farmers practising *Muang-fai*, a traditional communal irrigation system in Northern Thailand.
Type of data	Table Graph Image Excel file of raw data
How the data were acquired	A primary survey based on direct interviews was used to collect the data from 570 Longan farmers in 12 village locations in San Pa Tong District, Chiang Mai province, Northern Thailand (survey instrument is available as supplementary material). Half of the farmers practise the *Muang-fai* irrigation system. The location of each farm was also recorded using GPS.
Data format	Raw
Description of data collection	The survey's target frame was the group of Longan farmers who live within the 12 villages where *Muang fai* Sop Rong is located. This target group was sub-categorized into two: one group of *Muang fai* members and another group of non-members, who relied on pumping irrigation water from underground. Sampling was stratified by village, so that a similar number of members and non-members were randomly selected from each village.
Data source location	City: San pa thong Town: Mae Khong Klang Region: Northern Thailand Country: Thailand Latitude and longitude: 18.5837195, 98.9519292
Data accessibility	Mendeley [6]: Data (CSV): https://data.mendeley.com/public-files/datasets/t3zzhkxhf4/files/b7c9effb-10a6-48d2-9122-30c03a7a2771/file_downloaded
	Data (Stata): https://data.mendeley.com/public-files/datasets/t3zzhkxhf4/files/e6fae17f-3a05-4603-9acc-7b3312a734e8/file_downloaded Questionaire (Excel): https://data.mendeley.com/public-files/datasets/t3zzhkxhf4/files/7d3291f8-6bd0-4484-9f20-92d446cdaf85/file_downloaded
Related research article	Mungsunti, A., & Parton, K. A. (2017). Estimating the economic and environmental benefits of a traditional communal water irrigation system: The case of *Muang fai* in Northern Thailand. *Agricultural Water Management, 179*, 366–377 [2]. Mungsunti, A., & Parton, K. A. (2019). The sustainability of the *Muang fai* irrigation system of Northern Thailand. *AIMS Environmental Science, 6*(2), 77–93 [4]. Mungsunti, A., & Parton, K. A. (2021). The Price of Sustainability of a Traditional Irrigation System in Northern Thailand. *Sustainability, 13*(3), 1375 [5].

**Value of the Data**

• These data contain comprehensive information about farming, irrigation, exact location and socio-economic characteristics of farmers belonging to a traditional communal irrigation system called *Muang fai*, practised for centuries in Northern Thailand. The *Muang fai* is governed by a set of pre-established rules which, to a large extent, resemble Ostrom's [1] well-known principles of effective common property resources (see [2]). These data will be a useful addition to documentation of such practices.
• These data can benefit researchers (agricultural scientists, economists, geographers, historians, or social scientists in general) who study various types of small-scale irrigation, particularly traditional ones.
• These data can be used in further studies that compare various irrigation types. This includes comparing by scale of the system, by modern-vs-traditional, by country of location, or by rule or irrigation management. Additionally, any studies documenting long-traditions of irrigation may benefit from the public availability of these data. The exact geo-location of this dataset can also extend the benefit of this data to spatially-specific studies such as the effect of agglomeration or urbanization on the preservation of traditional irrigation management.
• In terms of its local policy context, the dataset also has importance and relevance. This research region is known for its Ching Mai and Lumphun Longan cultivation. Drought is increasingly an annual occurrence in these two provinces due to two reasons. First, water consumption has been rising due to the increased population, agricultural areas, and tourism activities. Second, the area's geography does not lend itself to water storage. As confirmed by Mungsunti and Parton [2] the *Muang-fai* system has an advantage over its competing practice (groundwater) in terms of water use efficiency.
• Relevant recent local policy development includes a new initiative Water where The Office of National Water Resources (ONWR) was directed in 2017 to collaborate with other relevant offices from 2017 to 2022, followed by a 20-year water management road map (any water crisis area or watershed must have a systemic moderate flooding plan and strategy).^1^

## 1 Data Description

**Fig. 1:**
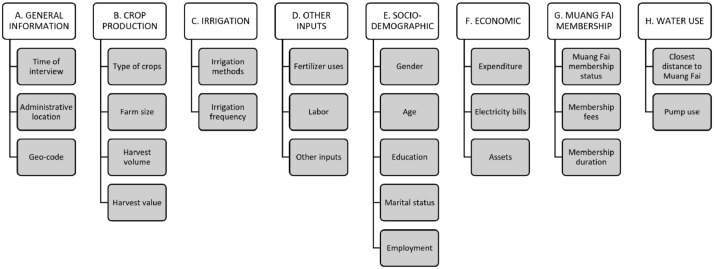
Summary of the content of the dataset.

The raw data in file mungfai.dta (Stata file) and mungfai.csv (Excel, comma-separated file) contain the answers from 570 farmers surveyed using the survey instrument/questionnaire (questionaire_EngThai_9_18.03.11.xlsx). The questionnaire contains general information (time of interview, location, including geo-coordinates), crop production, irrigation information, inputs used in farming, socio-economic and demographic conditions, status of *Muang fai* membership, and irrigation water usage. The questionnaire is available in both the local language (Thai) as well as in English [6]. The structure of the variables in the dataset is illustrated in Fig. 1.

Below is the detailed description or the definition of each of the variables collected in the survey as illustrated in Fig. 1.

(A) General information
  (1) Time of interview
    This contains the time and the date of the first and second (if needed) interview.
  (2) Administrative location
    This contains the name of the sub-district, village, and the assigned household sampling number.
  (3) Geo-code
    This contains the latitude and altitude of the farm location. This information is required to calculate the closest distance to the *Muang fai* canal. The data distributed with this paper has removed the geo code for privacy reasons.
(B) Crop production
  (1) Type of crops
    This contains the type (such as the name) of the crops the farmer grows including crops other than Longan (the focus of the study). Some farmers practise multi-crops farming in the same land plot. This survey also records such practices.
  (2) Farm size
    This variable contains the information on the size of the farm land (measured in *rais*, unit measurement of land in local language).
  (3) Harvest volume
    This contains information on the quantity (in kg) and quality (reflected by the average sales price in Baht/kg) of the harvest. Typically, the price per kilogram of Longan is proportional to the size of the fruit. Larger fruits can be sold at a higher price per kg.
(C) Irrigation methods
  This contains information on the type of irrigation used by the farmer. There are two types of irrigation namely using water from *Muang fai* canal or using underground water.
(D) Irrigation frequency
  The farmers were asked about the irrigation duration (in months, weeks, and days) during the growing season. These variables are required for estimating the amount of water (in cubic meters) used in the irrigation system.
(E) Other inputs
  (1) Fertilizer uses
    This contains information on how much (in kg) fertilizer was used during a growing season.
  (2) Labor
    This contains information on the number of laborers (other than the farmer) employed on the farm. It also tracks the number of days worked by laborers during each growing season. When combined with the wages farmers pay their employees, these data can be used to determine the operational costs of farming.
  (3) Other inputs
    This is an open question where respondents can list (up to three) inputs other than specified before.
(F) Socio-demographics
  (1) Gender
    This variable contains the information on the gender of the farmer i.e., whether the farmer was male or female.
  (2) Age
    This variable contains data on how old the farmer was at the time of the interview. When combined with the date the farmer began working as a farmer, age can reflect farming experience.
  (3) Education
    This variable records the education level of farmers. It has 5 education level categories namely whether the farmer (a) Not pass elementary school; (b) Has finished elementary; (c) Completed junior secondary school; (d) Completed senior secondary school; (e) Completed college/university.
  (4) Marital status
    This variable records the marital status of the farmers i.e., whether the farmer was married or single at the time of the interview.
  (5) Employment
    This contains the employment status of the farmer as well as all other family members. Employment status may be one of the following: (1) Working off-farm; (2) Not working off-farm. Number of days per week of the off-farm employment was also recorded during the interview.
(G) Economic
  (1) Expenditure
    This information indicates how much a household spends every week on consumption (in Thai Baht/week). This comprises food and non-food expenses incurred by the household (farmers and family members) during the preceding week.
  (2) Electricity bills
    This data records how much the household pays for the total electricity bills (in Thai Baht/month).
  (3) Assets
    This contains information on the ownership of such assets as cars, motor bikes, bicycles, televisions and livestock.
(H) *Muang fai* membership
  (1) *Muang fai* membership status
    This variable contains information on whether the farmer was a member of the *Muang fai* irrigation system.
  (2) Membership fees
    This variable records the fees that the farmer pays to become a member.
  (3) Membership duration
    This data records how long the farmer has been a member.
(I) Water use
  (1) Closest distance to *Muang fai* canal
    This is computed using GIS by combining the farm's latitude and longitude with the map of the *Muang fai* irrigation system. While interviewing the farmers, the enumerator used GPS to record the geolocation of each farm. The irrigation system map was created using satellite imagery provided from the Chiang Mai University Geography Department.
  (2) Pump use
    This section of the dataset comprises variables that record the usage of water pumps for irrigational purposes, including (a) whether the pump used electricity or gasoline; (b) the power of the electricity used; and (c) the horsepower of the gasoline used (in watts and horsepower). This information, along with the frequency of irrigation, can be used to determine the volume (in cubic meters) of water consumed during irrigation. The estimation of water efficiency is dependent on these variables.

Table 1 shows the summary of the variables listed above comparing members of the *Muang fai* irrigation and farmers who use underground water.

**Table 1:** Summary statistics of members of *Muang fai* and non-member/underground water users.

	*Muang fai* members	Underground water users
Number of samples	308	262

**Crop production**		
Growing crops other than Longan (%)	21.43	8.78
Farm size of main crops/longan (rais)	4.91	4.55
Productivity (kg/rai)	1125.76	954.88
Productivity (Thai Baht/rai)	20,257.94	15,318.96

**Irrigation**		
Water use (Meter cubic/rai)	3470.06	6220.55
Water use (Meter cubic/kg of harvest)	6.73	13.85
Water use (Meter cubic/Thai Baht sales of harvest)	0.39	0.85

**Other inputs**		
Fertilizer use (kg/rai in a growing season)	1851.90	1804.72
Number of farm workers	6.02	6.32

**Socio-demographics**		
Proportion of male farmers (%)	83.12	79.01
Age of farmers (years)	59.25	58.65
Without elementary education (%)	2.60	2.29
With elementary education (%)	74.68	78.24
With junior secondary education (%)	8.44	5.73
With senior secondary education (%)	9.09	8.40
With university/college education (%)	5.19	5.34
Proportion of married farmers (%)	88.31	87.02
Number of days of off-farm works (days)	2.02	2.48

**Economic**		
Expenditure per capita per month (Thai Baht)	666.33	766.72
Electricity bill (Thai Baht/month)	220.60	353.67

**Assets**		
Proportion of farmers that own a car (%)	59.74	58.78
Proportion of farmers that own a motorcycle (%)	95.78	93.51
Proportion of farmers that own a bicycle (%)	86.04	86.26
Proportion of farmers that own a television (%)	99.68	100.00
Proportion of farmers that own chicken(s) (%)	30.52	27.10

Source: Authors’ calculation.

From Table 1, we can highlight several observations. First, in general there is not much difference in characteristics between members and non-members of the *Muang fai*, except in the context of crop production and irrigation. For example, both *Muang fai* and non-*Muang fai* members have roughly the same average age (59.2 and 58.6 years old, respectively), have similar proportion of married farmers (88% and 87%, respectively), and have around 5% with college education. So, both seem to have similar socio-demographic characteristics.

Similarly, in terms of asset ownership, both show more similarities than differences. Among members of the *Muang fai*, for example, car ownership is 59.7% and among non-members it is 58.8%. Ownership of other types of assets also seemingly is quite similar.

The economic characteristics, particularly expenditure per capita per month, show little difference, where *Muang fai* members seem to have around a 15% smaller expenditure per capita than non-*Muang fai* members. Larger difference occurs in terms of electricity bills (almost 40% difference). However, this difference may be related to the use of electric pumps of underground water users.

Both groups (*Muang fai* members and non-*Muang fai* members) have also similar use of fertilizers and employ roughly the same number of laborers. However, in terms of value-productivity and water use efficiency, they are notably different. For example, farmers who are members of the *Muang fai* produce Longan worth of 20,257 Thai Baht per rai, while those who are non-Members produce Longan worth of 15,318 Thai Baht per rai, a 32.2% difference. Moreover, *Muang fai* members use irrigation water much more efficiently. While non-*Muang fai* members who use pumps to get underground water use 6220.5 m^3^ per rai for one growing season, *Muang-fai* members only use 56% of what non-*Muang fai* member use (3470.06 m^3^).

**Fig. 2:**
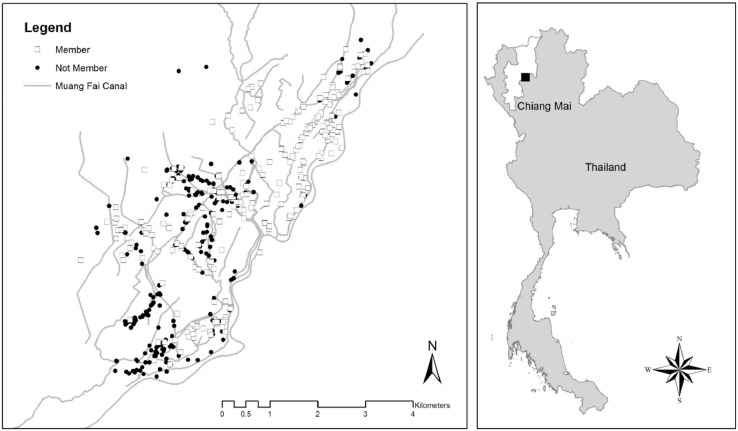
Study site location in the *Muang fai* Sop Rong irrigation area (first map and black square in the second map) (Source: [2]).

To illustrate more about the usefulness of the data, Fig. 2 (from [2]) shows the location of the survey including the locations of the respondents overlayed with the network of the *Muang fai* irrigation system in the area.

**Fig. 3:**
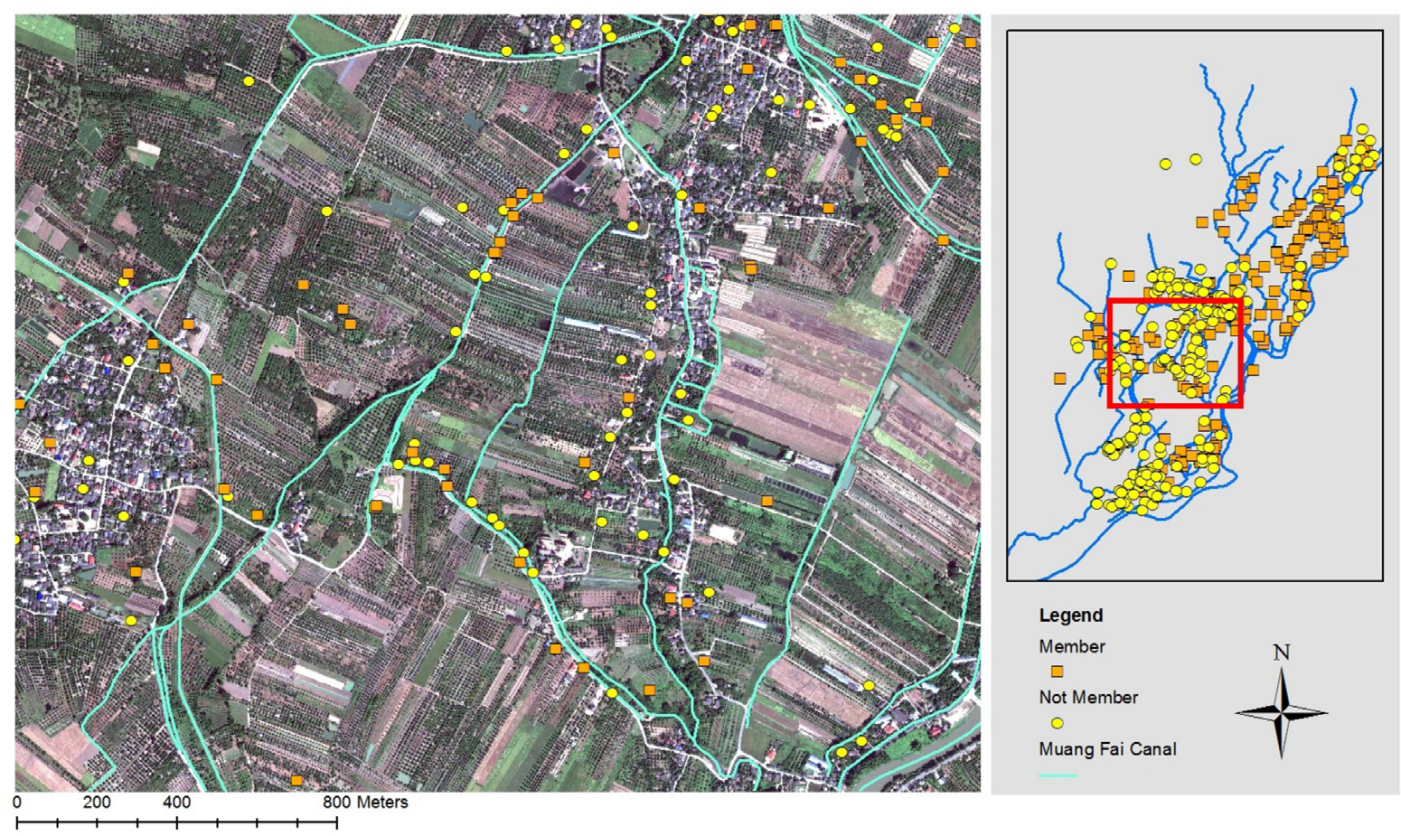
The study area from aerial photography (Source: [2]).

The map including the information from the survey can also be combined with other maps such as satellite imagery that may include, among others, land use of the area, such as illustrated in Fig. 3 (also from [2]).

## 2 Experimental Design, Materials and Methods

This case study's research area is in Thailand's northern region. It is situated at the northwest side of the province of Chiang Mai (as shown in Fig. 1). It is called the *Muang fai* Sop Rong, and is on the right bank of the Ping River in San Pa Tong District. For the local farmers who live in this region, the *Muang fai* Sop Rong is crucial. Covering about 937 ha of land, it is a significant source of water for the region's water needs, particularly for agricultural development. The system provides water through its main canal, which is 7.8 km long and runs through 12 villages in the San Pa Tong District [2,3]. The National Statistical Office [7] provides additional useful information on the region.

The *Muang fai* Song Rop was selected as the case study for a number of reasons. First and foremost, the ancient method of building irrigation systems, including some specific physical characteristics of traditional headwork construction from local materials like wood, logs, and stones, is still in use today. Second, a comparative analysis of this example is appropriate because many farmers in this region use ground water as an alternative irrigation method to *Muang fai*.

The group of Longan farmers was chosen as the survey's target population. We conducted a survey of farmers practising Longan farming in the *Muang fai* Sop Rong. The *Muang fai* members group and the ground water irrigation users group were separated into two groups of comparable size. The sampling was then divided into villages. From each village, we randomly picked two groups of members and non-members of roughly equal size.^2^

Prior to starting the survey, we had Focus Group Discussions (FGD) to help us refine the survey's questionnaire. We asked relevant parties, in particular neighborhood farmers, to help us improve the survey questionnaires. Additionally, some significant relevant informants were invited to take part in this FGD and to be interviewed. There were a number of informants who had first-hand experience of the study area, including the case study area's village chiefs, former *Muang fai* irrigation managers, regional irrigation specialists, and local administrative office workers. We began training the enumerators once we improved the questionnaire (who mostly were students from Chiang Mai University).

In order to reduce potential errors, we pre-tested the questionnaire before the survey. The survey was administered in the native Thai language. The pre-test survey was carried out by 14 students who were designated as enumerators using the draft survey questionnaire. In order to begin the pre-test survey, we gave each survey enumerator the task of interviewing 15 Longan farmers who lived nearby *Muang fai* Sop Rong. The actual survey was conducted in the months of March, April, and May 2011 after the pre-test survey was completed.

For this study, to measure the volume of irrigation water use, we used indirect methods, as described in [2]. In principle, the method records the frequency of irrigation from the interview as well as the capacity of the pump the farmers use. Using a smaller set of observations we established a statistical relationship between pump capacity and the volume of water irrigated to the farm land. We applied this relationships to all samples.

## Ethics Statements

We, hereby, confirm that relevant informed consent was obtained from all respondents in the survey. The research and its survey has been reviewed and approved, in accordance with the Australian National Health and Medical Research Council's National Statement on Ethical Conduct in Research Involving Humans, by Charles Sturt University (CSU) Human Research Ethics Committee, with issued protocol number 2011/033.

## CRediT authorship contribution statement

**Arriya Mungsunti:** Conceptualization, Methodology, Software, Data curation, Writing – original draft. **Kevin A. Parton:** Conceptualization, Methodology, Software, Writing – original draft. **Arief Anshory Yusuf:** Writing – original draft. **Tossapond Kewprasopsak:** Data curation.

## Declaration of Competing Interest

We declare that we have no known competing financial interests or personal relationships that could have appeared to influence the work reported in this paper.

## Data Availability

Questionnaire (Original data) (Mendeley Data). Survey Results (Original data) (Mendeley Data). Questionnaire (Reference data) (Mendeley Data).

## Footnotes

2 Several steps in the stratified random sampling technique were used to select sample farm households. Villages were the strata defined in this sampling, so that the final size of the sampling from each village was proportional to the number of Longan farmers from each village. Within each village (strata) the respondents were randomly selected to be interviewed. This process produced a probabilistically selected sample of 570 households, composed of 300 farmers who were members of the system and 270 farmers who were not [8].

## Footnotes

1 Based on the report: Consultant of technology company limited, Southeast Asia Technology company limited, and InfraTrans Consultants company limited. (n.a.), Documents for the project orientation meeting -Integrated Master Plan Study Project for Flood and Drought Mitigation area based Chiang Mai - Lamphun, Office of the National Water Resources. Link: http://1.179.162.22/myfile//เอกสารประกอบการประชุมปฐมนิเทศ%20.pdf
